# Experimental Scedosporiosis Induces Cerebral Oedema Associated with Abscess regarding Aquaporin-4 and Nrf-2 Depletions

**DOI:** 10.1155/2019/6076571

**Published:** 2019-04-04

**Authors:** Sumate Ampawong, Natthanej Luplertlop

**Affiliations:** ^1^Department of Tropical Pathology, Faculty of Tropical Medicine, Mahidol University, 420/6 Ratchawithi Road, Ratchathewi, Bangkok 10400, Thailand; ^2^Department of Microbiology and Immunology, Faculty of Tropical Medicine, Mahidol University, 420/6 Ratchawithi Road, Ratchathewi, Bangkok 10400, Thailand

## Abstract

Cerebral involvement especially brain abscess is life-threatening complication and major cause of death during* Scedosporium apiospermum* infection. However, little is known about pathogenesis of brain oedema associated with abscess in scedosporiosis. Experimental scedosporiosis was conducted in BALB/cMlac mice to characterize the presence of brain oedema, its type, and its related mechanisms focusing on aquaporin (AQP)-4, nuclear factor erythroid-2 related factor (Nrf-2), and tumor necrotic factor (TNF)-*α*. The results revealed that* S. apiospermum* infection induced severe inflammatory environment relevant to TNF-*α* expression and cytogenic oedema-associated brain abscess predominately in cerebrum of immunocompromised mice without voriconazole treatment reflecting to downregulation of AQP-4 in neighboring abscess areas and oedematous blood vessels. Downregulation of Nrf-2 in neuronal cells and myelin degeneration were significantly observed in nontreated mice. In summary, oxidative stress, severe inflammatory response, and space-occupying mass from abscess formation inducing tissue hypoxia might be the postulate causes of oedema induced by scedosporiosis.

## 1. Introduction

Scedosporiosis, an opportunistic fungal infection caused by* Pseudallescheria boydii *species complex, leads to several kinds of life-threatening symptoms involving the central nervous system (CNS) and lung complications [1, 2]. Important risk populations for CNS infection are either in near-drowning immunocompetent victims or in medical immunocompromised patients due to organ transplantation, chemotherapy, radiotherapy, and immunosuppressive diseases, e.g., diabetes mellitus, hepatitis, anemia, and AIDS. Hallmark histopathological findings in CNS are abscess, meningitis, meningoencephalitis, cerebral aneurysm, and diffused hemorrhage. Singular and multiple abscesses are frequently found in the cerebral areas particularly in frontal, parietal, and temporal lobes [2]. Recently, immunopathogenesis of scedosporiosis was mainly focused on (i) innate immune response to provide oxidative respiratory burst and antimicrobial enzymes for elimination of fungal compartment under an oxidative stress environment and (ii) tissue reaction leading to the abscess formation for limitation of fungal distribution [3]. In addition, fungal elements from* Scedosporium spp. *especially cell wall can trigger macrophage to release tumor necrotic factor (TNF)-*α* resulting in severe host inflammatory response [4].

It has been well recognized for decades that aquaporin (AQP)-4, water homeostasis membrane protein, plays important role in the formation of brain oedema and closely relates to blood brain barrier (BBB) configuration in several conditions, e.g., rodent cerebral malaria [5], hypoxia-induced brain oedema [6], brain tumor-associated septic encephalopathy [7], and staphylococcal brain abscess [8]. Considering the pathogenesis of brain oedema induced by cerebral abscess, not only directly caused by the infectious organisms, host immune response is also mainly characterized by the substantial oedema neighboring abscess and increases BBB permeability due to proinflammatory cytokines from the abscess [8, 9]. Unfortunately, brain oedema associated with cerebral abscess in* Scedosporium apiospermum* infection under oxidative and inflammatory environment regarding AQP-4 and nuclear factor erythroid-2 related factor (Nrf-2), a master transcription factor for antioxidant response, has not been yet well studied.

Therefore,* in vivo* model of scedosporiosis was conducted in the present study, to investigate the role of AQP-4 and Nrf-2 in the brain oedema-associated abscess using histopathological, immunohistochemical, and electron microscopic studies. This study provides a better understanding of the pathogenesis of the oedematous formation in brain abscess-induced by* S. apiospermum* infection leading to the new target for alleviation of the life-threatening complications in scedosporiosis.

## 2. Materials and Methods

### 2.1. Ethical Statement

This research project was conducted under the “Animal for Scientific Purposes Act, B.E. 2558 (A.D. 2015), Thailand”. All animal experiments were approved by Institutional Animal Care and Use Committee of Khon Kaen University (approval number: 0514.1.75/34).

### 2.2. Fungal Strain and Preparation

*S. apiospermum* was plated on Scedo-Select III medium; the plates were incubated for 7 days at 32°C. Conidia had been collected by washing with sterile phosphate-buffered saline (PBS, pH 7.2) and adjusted to a concentration of 10^6^ conidia/ml.

### 2.3. Experimental Scedosporiosis and Treatment

Scedosporiosis mouse model was conducted in 8-weight old, female BALB/cMlac mice, purchased from National Laboratory Animal Center, Mahidol University. Consequent to a quarantine period, neutropenia was induced in all mice by intraperitoneal injection of 200 mg/kg cyclophosphamide [10–12]. Leukocyte count was performed 1 day after induction to confirm neutropenic stage. Neutropenic mice were intravenously injected with 10^6^ conidia of* S. apiospermum*. Twenty-four hours after infection, mice were randomly separated into two groups, with or without treatment. Treated mice were intraperitoneally injected with 20 mg/kg/day of voriconazole (Sigma®, Germany) while nontreated group received 200-*μ*l of 5% dextrose in 0.9% sodium chloride (D5S)/day. The clinical manifestation either death or moribund was carefully observed every day until 7 days after infection. The number of survival and death of mice was also recorded. The moribund mice were humanely euthanized with overdose of isoflurane inhalation.

### 2.4. Histopathological Study

Brains were dissected and fixed in 10% neutral buffer formalin for 48 hr. All tissues were dehydrated, infiltrated, and embedded under standard tissue processing. The specimens were cut into 5-*μ*m thickness and stained with Hematoxylin & Eosin. Histopathological changes in the brain were examined under light microscope focusing on brain abscess or microabscess, perivascular cuffing with white blood cells, perivascular oedema, meningitis, and hemorrhage. The severity of each mentioned histological change was scored into four grades: 0=absent, 1=mild, 2=moderate, and 3=severe. Histopathological score, ranging from 0 to 18, was calculated by the combination of each histological change.

### 2.5. Immunohistochemical Study

To determine the presence of brain oedema in both oxidative stress and inflammation environments causing by* S. apiospermum* infection, cerebral aquaporin (AQP)-4, nuclear factor erythroid-2 related factor (Nrf-2), and tumor necrotic factor (TNF)-*α* expressions were evaluated, respectively, using immunohistochemical staining as mentioned in the previous studies [5, 13]. Polyclonal rabbit anti-AQP-4, -Nrf-2, and -TNF-*α* (MyBiosource®, USA) were used as a primary antibody and applied to the tissues consequence to heat-retrieved antigenicity in citrate buffer, pH 6.0. The tissue was then incubated with EnVision FLEX/HRP (K8002; DAKO, Denmark), visualized by DAB, and counterstained by Hematoxylin. Immunohistochemical study was examined under light microscope.

AQP-4 semiquantitation was performed in the brain parenchymal area and blood vessel using image analysis program (ImageJ, version 1.51J8, NIH). Ten images of each parenchyma (cerebrum, hippocampus, brain stem, olfactory bulb, diencephalon, and cerebellum) and blood vessel in all mice were acquired by light microscope (BX41, Olympus®, Japan) and digital camera (DP20, Olympus®, Japan) at 400X magnification. Colour images were transferred to binary images. The area of expression was located using threshold adjustment and measured into percentage of area fraction (expression)/image. The perivascular and vascular expressions of AQP-4 were examined by drawing the straight line over either perivascular or vascular area to locate an area of expression. AQP-4 labeling was measured as percent/perivascular or vascular area as previously mentioned. In addition, the level of Nrf-2 and TNF-*α* in the brain was also explored by counting the number of positive cells/high power field (40X). The counting was conducted 10 filed/mice.

### 2.6. Electron Microscopic Study

To confirm the presence of cerebral oedema and other ultrastructural changes in association with* S. apiospermum* infection, fine morphological study was conducted. Dissected brains were cut into 2x2 mm, primary fixed in 2.5% glutaraldehyde in 0.1 M sucrose phosphate buffer (SPB) and secondary fixed in 1% osmium tetroxide in SPB. The tissues were dehydrated in a series of ethanol, infiltrated in grading LR white resin (EMS, USA), embedded in pure LR white, cured in 65°C oven, and sectioned into 100 nm thick. The sections were stained with uranyl acetate and lead citrate and examined under transmission electron microscope (HT7700; Hitachi, Japan) focusing on perivascular oedema, astrocytic foot process, and myelin conformation to determine the presence of vasogenic or cytogenic oedema and number of myelin degeneration/field (at 2,000X magnification).

### 2.7. Statistical Analysis

GraphPad® PRISM, version 6.05, was used for statistical analysis. Either nonparametric of independent t-test or analysis of variance (ANOVA) was performed to differentiate the difference between groups. Log-rank (Mantel-Cox) test was used to determine the survival time between treated or nontreated group. The significant level was considered at p value < 0.05.

## 3. Results

### 3.1. Survival Time

**Figure 1:**
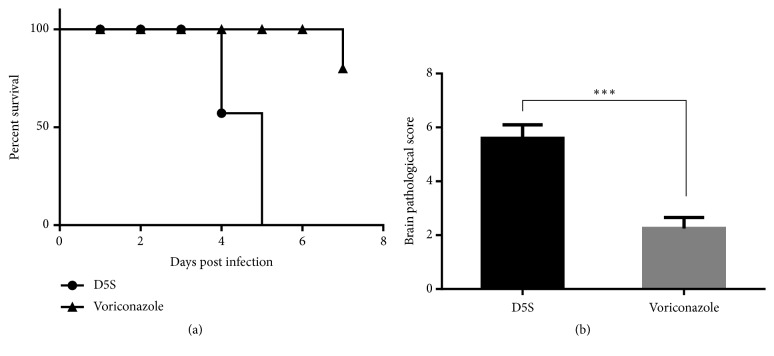
*Survival rate and brain histopathological score*. The comparison of survival times during postinoculation periods between rats with or without treatment (a). Brain histopathological score from those rats was compared (b). *∗∗∗*: p value < 0.0001.

After inoculation with* S. apiospermum* for 2-5 days, severe neurological symptoms, moribund, and death were observed in nontreated mice. All of them died within 5 days after inoculation, while 10% (1/10) of voriconazole-treated mice died on day 7 after inoculation. The survival time in nontreated mice (2.72 ± 0.281 days) was significantly lowered than treated mice (5.83 ± 0.343 days) (Figure 1(a)).

### 3.2. Histopathological Study

**Figure 2:**
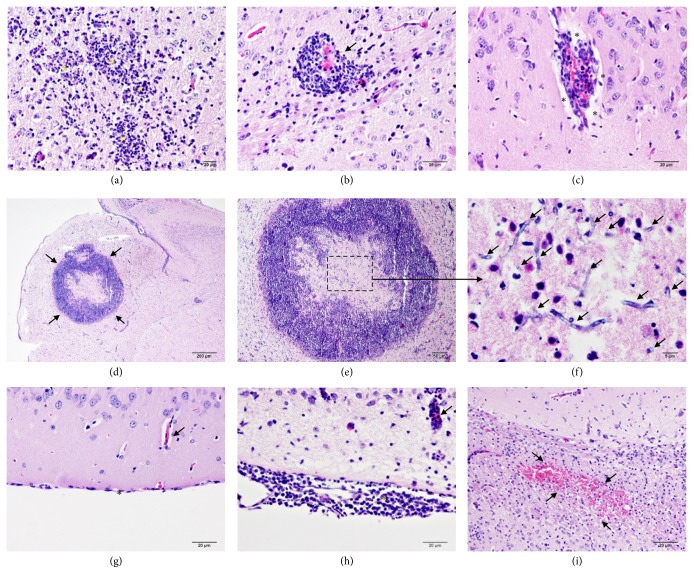
*Brain histopathological findings in scedosporiosis rats with or without treatment*. After inoculation with* Scedosporium apiospermum* for week, two stages of brain abscess were developed, early ((a)-(c)) and late ((d)-(e)) cerebritis. In early cerebritis, perivascular cuffing mainly with neutrophil was found around the site of infection (*∗*). The appearances of perivascular cuffing were characterized by either absent (b) or present (c) of the perivascular space (*∗*). In late cerebritis, a granulomatous lesion was observed ((d)-(e)). This lesion composed of a central necrotic area, where a number of fungal hyphae were found ((f); arrow), surrounded with a pack of inflammatory cells particularly neutrophil without fibrous capsule formation. Unlike the brain without abscess (g), the presence of mononuclear cells deposition in the meninges was noted in the abscessed brain ((h); *∗*). Moreover, severe hemorrhage was generally seen in cerebral parenchyma ((i); arrow).

*S. apiospermum* infection without treatment induced severe histopathological changes in the brain when compared to voriconazole-treated mice as presented in Figure 1(b), ranging from perivascular cuffing, microabscess, perivascular area distension, brain abscess surrounding oedema, meningitis, and hemorrhage (Figure 2). Two different stages of brain abscess were found in all mice without treatment, while only one mouse with voriconazole treatment exhibited brain abscess. In addition, the number of brain abscess was ragingly observed in cerebral cortex, hippocampus, and brain stem, respectively. However, olfactory bulb, diencephalon, and cerebellum were not presented any abscess.

### 3.3. Immunohistochemical Study

**Figure 3:**
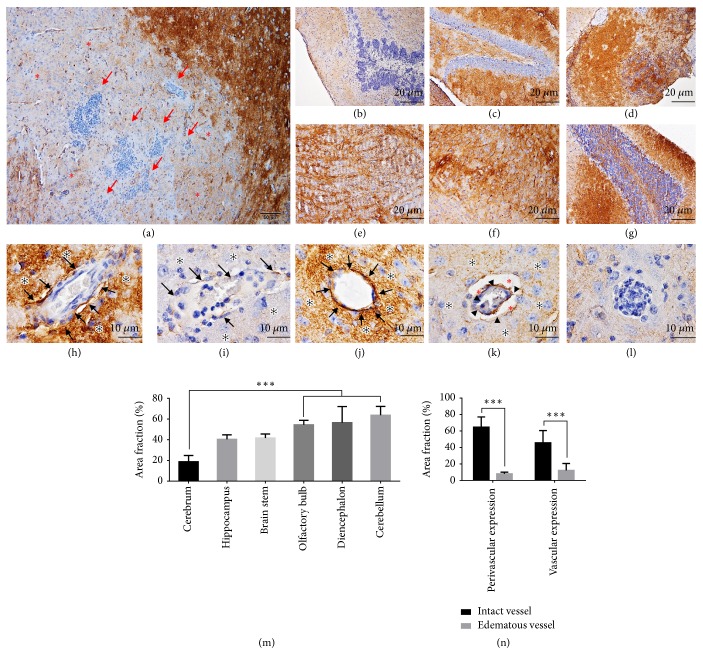
*Aquaporin-4 expression in the brain*. Immunohistochemical micrographs of AQP-4 labeling in several parts of the brain; cerebrum ((a)-(b)), hippocampus (c), olfactory bulb (d), brain stem (e), diencephalon (f), and cerebellum (g) and in either perivascular area ((h)-(k); *∗*) or its blood vessel ((h)-(k); arrow) as presented by intact ((h) and (j)) or oedematous blood vessel ((i) and (k)). The expression of AQP-4 in the microabscess and percentage of its expression among cerebral areas, perivascular area, and cerebral blood vessel were also demonstrated in (l) and compared in (m) and (n), respectively. AQP-4 immunolocalization in cerebrum was significantly lower than that presented in olfactory bulb, diencephalon, and cerebellum (m). The oedematous vessel also exhibited lower AQP4 expression than intact vessel in both perivascular and vascular areas (n). *∗∗∗*: p value < 0.0001.

AQP-4, water channel membrane protein, normally is expressed on the cerebral endothelial and neuronal cells to regulate water movement in the brain [14–17]. Immunohistochemical study revealed that downregulation of AQP-4 was found in either neighboring area of brain abscess or oedematous blood vessel (Figure 3). The level of AQP-4 expression was significantly decreased in cerebrum when compared to olfactory bulb, diencephalon, and cerebellum (Figure 3(m)). Perivascular and vascular expressions of AQP-4 in oedematous blood vessels were significantly lowered than the intact one (Figure 3(n)).

**Figure 4:**
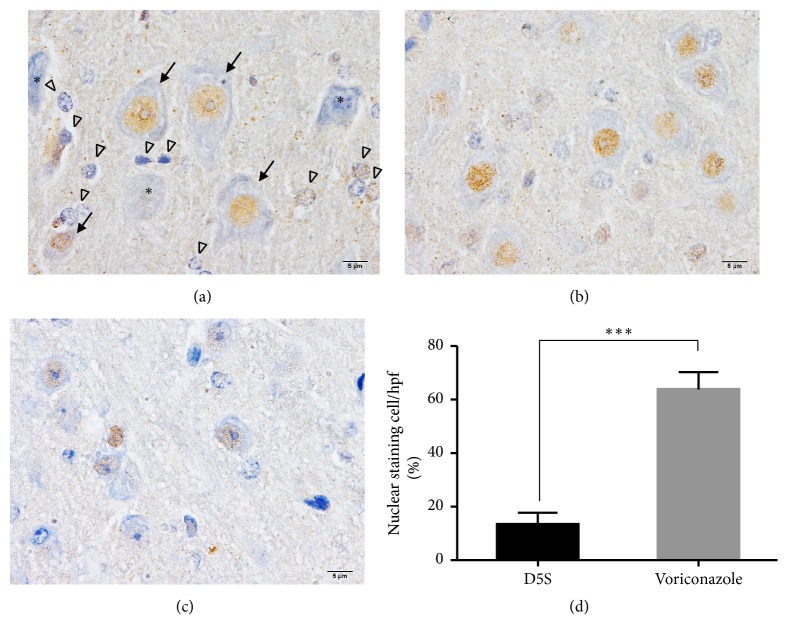
*Immunohistochemical staining of Nrf-2 in the neurons*. Nrf-2 labeling was expressed in the nucleus of the neuronal ((a); arrow) and supporting ((a); arrowhead) cells. However, the nucleolar expression was decreased in some of the abnormal neurons ((a); *∗*). The comparison of Nrf-2 level between the brain without (b) or with (c) treatment was shown in (d). *∗∗∗*: p value < 0.0001.

**Figure 5:**
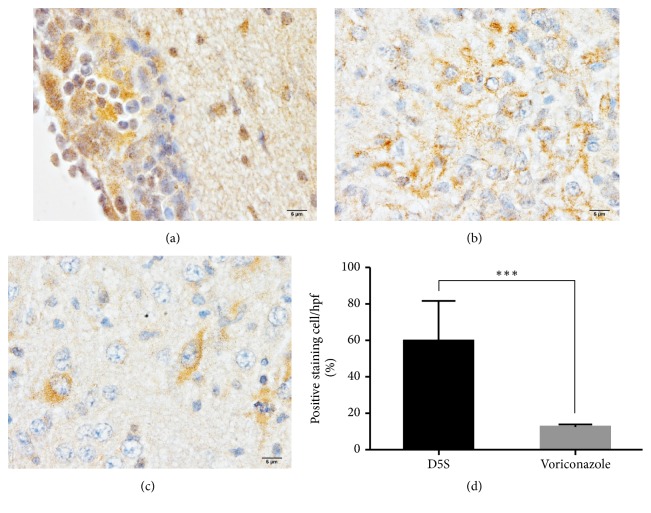
*The level of TNF-α in the brain with or without abscess*. The labeling of TNF-*α* in abscessed brain was intensely expressed in the meningeal areas where were deposited of inflammatory cells leading to meningitis (a). In the parenchymal area, higher expression of TNF-*α* was found in the brain with abscess (b) when compared to nonabscessed brain (c). The comparison of positive staining cell between treated or nontreated rats was compared (d). *∗∗∗*: p value < 0.0001.

To verify the oxidative stress and inflammation environments during* S. apiospermum* infection between treated and nontreated conditions, Nrf-2 and TNF-*α* labeling were examined, respectively. Downregulation of Nrf-2 and upregulation of TNF-*α* were demonstrated in nontreated mice as shown in Figures 4 and 5, respectively.

### 3.4. Transmission Electron Microscopic Study

**Figure 6:**
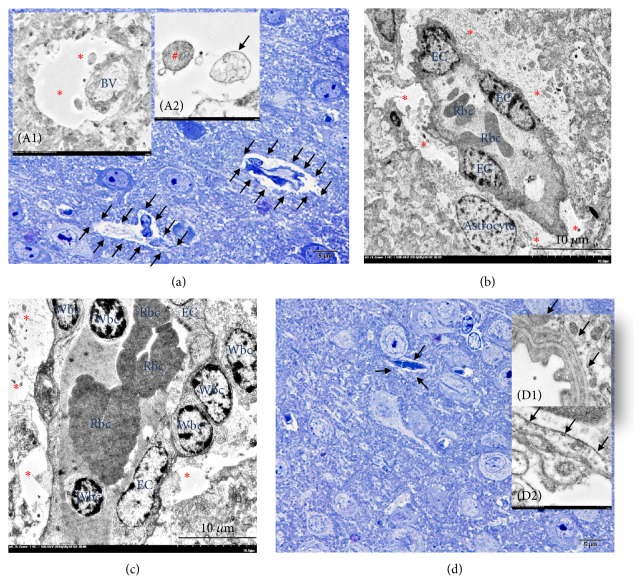
*Ultrastructure of cytogenic oedema in scedosporiosis-induced cerebral abscess*. In the brain with abscess, the presence of cytogenic oedema was frequent. It was characterized by astrocyte foot process dilatation where the perivascular area was distended ((a), (b) and (c); arrow and (A1); *∗*) and contained with intracellular materials such as mitochondria ((A2); #) or multivesicular bodies ((A2); arrow). In the brain without abscess, the presence of the perivascular area was less than in abscessed brain. The perivascular area ((d); arrow) and the endothelial lining cell were intact ((D1); arrow) when compared to the brain with abscess represented by vacuolated endothelial cell ((D2); arrow).

**Figure 7:**
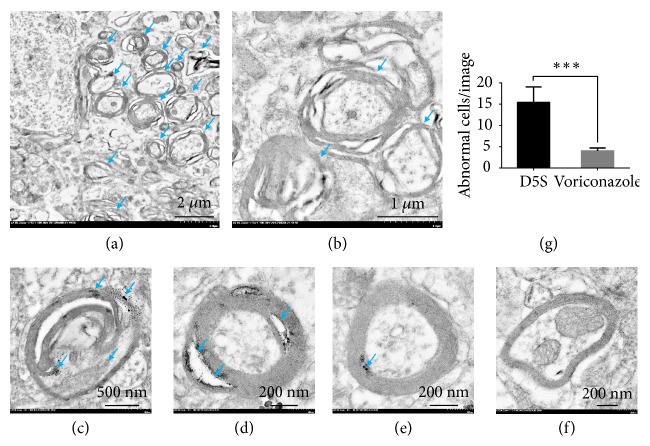
*Myelin damage in scedosporiosis-induced cerebral abscess*, The ultrastructural conformation of the myelin sheath was markedly lost in nontreated rats as presented by disorganization and swollen of the myelin sheath ((a)-(b); arrow, low and higher magnifications, respectively). High level of myelin damage was shown in (c)-(d) when compared to mild defect (e) and intact (f) myelinated nerves. The severity of myelin degeneration was compared in (g). *∗∗∗*: p value < 0.0001.

Generally, there are two types of oedema, cytogenic and vasogenic oedema, categorized by the severity stages of water leakage in association with BBB (composes of endothelial cell, basement membrane, and astrocyte foot process) conformation [18]. To clarify the type of oedema occurring in* S. apiospermum* infection, ultrastructural study was performed. The results exhibited that cytogenic oedema with endothelial cell degeneration, e.g., vacuolation, was pronouncedly found in the abscessed brain (Figure 6). Moreover, myelin degeneration in nontreated mice was also higher presented than in voriconazole-treated group (Figure 7).

## 4. Discussion

CNS infection by* S. apiospermum* in immunocompromised human cases was firstly reported by Benham et al. [19] represented by solitary or diffused brain abscess particularly in cerebrum and cerebellum, meningoencephalitis, ventriculitis, hemorrhage, and cerebral aneurysm [1]. To investigate the pathogenesis and develop new treatment for* S. apiospermum* infection, experimental scedosporiosis has been established for decades [10, 12, 20]. In agreement with the previous reports, the present study exhibited similar mortality rate, survival time, and histopathological changes. A hundred percent lethality was completely found on 5 days after infection of 10^6^ conidia in nontreated immunosuppressive mice (Figure 1). Histopathological findings in the brain mainly composed perivascular cuffing, microabscess, brain abscess surrounding with oedematous area, meningitis, hemorrhage, and perivascular distension (Figure 2). However, the presence of perivascular area distension in association with brain oedema induced by* S. apiospermum* infection has not yet been reported and is still unclear.

Generally, brain abscess formation is considered as a space-occupying lesion, which is composed of several stages, either early or late of cerebritis and encapsulation. Perivascular cuffing with neutrophils at the site of infection, in addition to periphery edema, is the main pathology of early cerebritis. In late cerebritis, the central area becomes necrosis, while the outer is deposited with fibroblast to form fibrous connective tissue. Further stages are early and late encapsulations; well vascularization of the ring-enhancing capsules is developed. Reactive gliosis has also been found. Finally, a thickening capsule is effectively walled off the suppurative area. The present study demonstrated that* S. apiospermum* induced brain abscess within 2-5 days after infection predominately in immunocompromised nontreated mice. Early and late cerebritis with periphery oedema was developed without capsule formation (Figure 2). Interestingly, there is evidence that cerebritis-induced by infectious diseases, e.g.,* Staphylococcus aureus,* alters BBB permeability and causes brain oedema [8, 9]. Like other reports, therefore, a high possibility of brain oedema-associated abscess was exhibited in scedosporiosis.

In this study, we investigated the pathogenesis of brain oedema caused by* S. apiospermum* infection using water homeostasis marker (AQP-4) and ultrastructural changes, either astroglial dilatation or perivascular oedema as described in our previous study [5] in cooperation with the level of antioxidative (Nrf-2) and proinflammatory (TNF-*α*) markers. The results showed that AQP-4 expression in abscessed brain was decreased particularly in cerebrum where the microabscess and brain abscess were predominately found. The intact blood vessel and its surrounding parenchyma had strong expression of AQP-4 when compared to oedematous and perivascular cuffing blood vessels (Figure 3). As reported by Bloch et al. [8], they claimed that the increment of AQP-4 reduces the severity of brain oedema associated with cerebral infection. The decrease of AQP-4 in our study suggested a high possibility that excess water cannot be cleared from affected areas leading to brain oedema. In the opposite way, a higher level of AQP-4 in treated mice exhibited an adaptive response to balance water in or out flux during* S. apiospermum* infection without brain abscess formation under the treatment effect of voriconazole.

Electron microscopic study indicated that cytogenic oedema was highly presented in the abscessed brain-induced by scedosporiosis as characterized by astroglial dilatation as mentioned in our pervious study [5] (Figure 6). AQP-4 channel on the perivascular astrocyte promotes the water movement in between vascular space, endothelial cell, and neuronal cell. The imbalance of water flux is caused by the depletion of AQP-4 leading to an accumulation of water in the cell or cytogenic oedema [21]. In consonance to previous report, main postulates of water flux disturbance in scedosporiosis are (i) physical disruption due to space-occupying lesion from abscess formation resulting in high cerebral pressure and subsequent to neuronal ischemia and (ii) biochemical signal, e.g., proinflammatory cytokines especially TNF-*α* leading to permeability alteration. However, the detail mechanisms for water clearance in the abscessed brain caused by scedosporiosis need to be further studied.

According to Xisto et al. [4], during inflammatory response in* S. apiospermum* infection, TNF-*α* was upregulated in nontreated immunocompromised mice with cerebral abscess (Figure 5). These provided an oxidative stress environment in the brain parenchyma leading to downregulation of Nrf-2 in the abscessed brain as shown in Figure 4. Nrf-2 pathway contributes to a major regulator of antioxidative stress from several conditions especially inflammatory responses and enhances cytoprotective effect and cellular homeostasis [22], by regulating the phase II detoxifying enzymes, e.g., glutathione peroxidase and heme oxygenase-1 [23]. Interestingly, several studies have reported that Nrf-2 pathway has modulated effects on brain oedema [24] due to neuronal inflammation and injury [25–27]. The present study demonstrated that nontreated immunocompromised mice with oedematous brain abscess failed to control the level of oxidative stress due to the depletion of Nrf-2 in the brain. Moreover, the relationship between downregulation of AQP-4 and Nrf-2 under oedematous condition in the brain has been reported [28]. Nrf-2, acting as a transcriptional factor for cellular protective and antioxidative genes, plays a crucial role in the activation of AQP-4 expression in several neurological disorders especially brain injury in associated with oedema [29, 30]. Nrf-2 deficient mice showed the high severity of brain oedema in traumatic cerebral injury [31, 32]. Therefore, it has a high possibility of claim that there are very close correlation between the reduction of AQP-4 and Nrf-2 expressions during scedosporiosis-induced cerebral oedema.

Regarding the discrepancy between mouse brain abscess model and human brain abscess case, the successful response to voriconazole in scedosporiosis from human cases [33] is lower than observed in the animal study. It could be postulated that the difference degree of therapeutic responses might be from several factors. In human, some cases which got earlier treatment had a high chance to recover when compared to the one who had delayed diagnosis in contrast to the experimental model. Mouse model is likely to be a more acute phase of the formation of brain abscess and had more permeability of blood brain barrier compared to human cases. However, further studies need to be required for these postulations.

In addition, another remark of an ultrastructural change in the brain was myelin degeneration, highly presented in nontreated immunocompromised mice (Figure 7). A breakdown of myelin sheath is one of the causes of axonal degeneration, which closely reflects to brain oedema [34] and relates to the high intracranial pressure condition [35] and the release of proinflammatory cytokines, e.g., TNF-*α* [36]. In line with these evidences, brain oedema induced by* S. apiospermum* infection leads to myelin degeneration due to the cytokines from abscess, cerebral oedematous condition, and the increase of intracranial pressure from space-occupying mass.

## 5. Conclusions

In conclusion, several lines of evidence in our study indicated that scedosporiosis-induced brain oedema associated with abscess was caused by severe responses of host inflammatory reaction and the increment of intracranial pressure from abscess mass under inappropriate AQP-4 and Nrf-2 responses. Cytogenic oedema was profoundly observed in* S. apiospermum* infection in relation to the depletion of AQP-4 and Nrf-2. Brain oedema, space-occupying condition, and lacking of neuroprotective effect due to Nrf-2 reduction may deteriorate myelin conformation in experimental scedosporiosis.

## Data Availability

The data used to support the findings of this study are available from the corresponding author upon request.

## References

[1] Guarro J., Kantarcioglu A. S., Horré R. (2006). *Scedosporium apiospermum*: changing clinical spectrum of a therapy-refractory opportunist. *Medical Mycology*.

[2] Kantarcioglu A. S., Guarro J., De Hoog G. S. (2008). Central nervous system infections by members of the Pseudallescheria boydii species complex in healthy and immunocompromised hosts: epidemiology, clinical characteristics and outcome. *Mycoses*.

[3] Cortez K. J., Roilides E., Quiroz-Telles F. (2008). Infections caused by *Scedosporium spp*. *Clinical Microbiology Reviews*.

[4] Xisto M. I. D. S., Bittencourt V. C. B., Liporagi-Lopes L. C. (2015). O-Glycosylation in cell wall proteins in Scedosporium prolificans is critical for phagocytosis and inflammatory cytokines production by macrophages. *PLoS ONE*.

[5] Ampawong S., Combes V., Hunt N. H. (2011). Quantitation of brain edema and localisation of aquaporin 4 expression in relation to susceptibility to experimental cerebral malaria. *International Journal of Clinical and Experimental Pathology*.

[6] Wang B., Li W., Jin H. (2018). Curcumin attenuates chronic intermittent hypoxia-induced brain injuries by inhibiting AQP4 and p38 MAPK pathway. *Respiratory Physiology & Neurobiology*.

[7] Davies D. C. (2002). Blood-brain barrier breakdown in septic encephalopathy and brain tumours. *Journal of Anatomy*.

[8] Bloch O., Papadopoulos M. C., Manley G. T., Verkman A. S. (2005). Aquaporin-4 gene deletion in mice increases focal edema associated with staphylococcal brain abscess. *Journal of Neurochemistry*.

[9] Lo W. D., Wolny A., Boesel C. (1994). Blood-brain barrier permeability in staphylococcal cerebritis and early brain abscess. *Journal of Neurosurgery*.

[10] Gonzalez G. M., Tijerina R., Najvar L., Rinaldi M., Yeh I.-T., Graybill J. R. (2002). Experimental murine model of disseminated Pseudallescheria infection. *Medical Mycology*.

[11] Legrand C., Anaissie E., Hashem R., Nelson P., Bodey G. P., Ro J. (1991). Experimental fusarial hyalohyphomycosis in a murine model. *The Journal of Infectious Diseases*.

[12] Rodríguez M. M., Pastor F. J., Salas V., Calvo E., Mayayo E., Guarro J. (2010). Experimental murine scedosporiosis: histopathology and azole treatment. *Antimicrobial Agents and Chemotherapy*.

[13] Ampawong S., Isarangkul D., Aramwit P. (2017). Sericin improves heart and liver mitochondrial architecture in hypercholesterolaemic rats and maintains pancreatic and adrenal cell biosynthesis. *Experimental Cell Research*.

[14] Manley G. T., Hemphill J. C., Morabito D. (2000). Cerebral oxygenation during hemorrhagic shock: perils of hyperventilation and the therapeutic potential of hypoventilation. *Journal of Trauma*.

[15] Papadopoulos G., Sgouropoulou S., Arnaoutoglou E., Petrou A. (2002). Postoperative hypoxaemia in a patient with patent foramen ovale. *European Journal of Anaesthesiology*.

[16] Saadoun S., Papadopoulos M. C., Davies D. C., Krishna S., Bell B. A. (2002). Aquaporin-4 expression is increased in oedematous human brain tumours. *Journal of Neurology, Neurosurgery & Psychiatry*.

[17] Vajda Z., Pedersen M., Fuchtbauer E. M. (2002). Delayed onset of brain edema and mislocalization of aquaporin-4 in dystrophin-null transgenic mice. *Proceedings of the National Academy of Sciences of the United States of America*.

[18] Klatzo I. (1994). Evolution of brain edema concepts. *Acta neurochirurgica. Supplement*.

[19] Benham R. W., Georg L. K. (1948). Allescheria boydii, causative agent in a case of meningitis. *Journal of Investigative Dermatology*.

[20] Leliévre B., Legras P., Godon C. (2013). Experimental models of disseminated scedosporiosis with cerebral involvement. *The Journal of Pharmacology and Experimental Therapeutics*.

[21] Manley G. T., Fujimura M., Ma T. (2000). Aquaporin-4 deletion in mice reduces brain edema after acute water intoxication and ischemic stroke. *Nature Medicine*.

[22] Kansanen E., Kuosmanen S. M., Leinonen H., Levonenn A.-L. (2013). The Keap1-Nrf2 pathway: mechanisms of activation and dysregulation in cancer. *Redox Biology*.

[23] Ahmed S. M., Luo L., Namani A., Wang X. J., Tang X. (2017). Nrf2 signaling pathway: Pivotal roles in inflammation. *Biochimica et Biophysica Acta (BBA) - Molecular Basis of Disease*.

[24] Chen X., Wang H., Zhou M. (2018). Valproic acid attenuates traumatic brain injury-induced inflammation in vivo: involvement of autophagy and the Nrf2/ARE signaling pathway. *Frontiers in Molecular Neuroscience*.

[25] Dong W., Yang B., Wang L. (2018). Curcumin plays neuroprotective roles against traumatic brain injury partly via Nrf2 signaling. *Toxicology and Applied Pharmacology*.

[26] Li H., Wang P., Huang F. (2018). Astragaloside IV protects blood-brain barrier integrity from LPS-induced disruption via activating Nrf2 antioxidant signaling pathway in mice. *Toxicology and Applied Pharmacology*.

[27] Wei C., Kong Y., Li G., Guan Y., Wang P., Miao C. (2017). Nicotinamide mononucleotide attenuates brain injury after intracerebral hemorrhage by activating Nrf2/HO-1 signaling pathway. *Scientific Reports*.

[28] Umenishi F., Verkman A. S. (1998). Isolation and functional analysis of alternative promoters in the human aquaporin-4 water channel gene. *Genomics*.

[29] Zhao J., Moore A. N., Clifton G. L., Dash P. K. (2005). Sulforaphane enhances aquaporin-4 expression and decreases cerebral edema following traumatic brain injury. *Journal of Neuroscience Research*.

[30] Desai B., Hsu Y., Schneller B., Hobbs J. G., Mehta A. I., Linninger A. (2016). Hydrocephalus: The role of cerebral aquaporin-4 channels and computational modeling considerations of cerebrospinal fluid. *Neurosurgical Focus*.

[31] Jin W., Wang H., Yan W. (2009). Role of Nrf2 in protection against traumatic brain injury in mice. *Journal of Neurotrauma*.

[32] Jin W., Wang H.-D., Hu Z.-G., Yan W., Chen G., Yin H.-X. (2009). Transcription factor Nrf2 plays a pivotal role in protection against traumatic brain injury-induced acute intestinal mucosal injury in mice. *Journal of Surgical Research*.

[33] Troke P., Aguirrebengoa K., Arteaga C. (2008). Treatment of scedosporiosis with voriconazole: clinical experience with 107 patients. *Antimicrobial Agents and Chemotherapy*.

[34] Perez-Torres C. J., Yuan L., Schmidt R. E., Rich K. M., Ackerman J., Garbow J. R. (2015). Perilesional edema in radiation necrosis reflects axonal degeneration. *Journal of Radiation Oncology*.

[35] Barz H., Schreiber A., Barz U. (2017). Demyelinating diseases as a result of cerebral edema?. *Medical Hypotheses*.

[36] Lock C., Oksenberg J., Steinman L. (1999). The role of TNFalpha and lymphotoxin in demyelinating disease. *Annals of the Rheumatic Diseases*.

